# Minnesota as a Resort for Consumptives

**Published:** 1871-01

**Authors:** 


MINNESOTA AS A RESORT FOR CONSUMPTIVES, is the caption of a brief article by Dr. Adams, of Waltham, in which he shows, from meterological and other statistics, that the winters of Minnesota are about equal in temperature to those of northern New England, while its summers are as warm as in Central Pennsylvania, the mean of the former being 16 deg., and of the latter 70 deg., F. But in winter there is very little rainfall, (snow being measured as rain in the proportion of 12 to 1); hence the atmosphere is very dry, and with the low temperature, has very little conducting power of heat. To this dryness of atmosphere Minnesota owes its reputation as a resort for consumptives, and it is not wise to send such patients there until the late autumn, for its rainfall in summer is equal to that of New England, while its temperature is higher. It is during the cold and dry season that phthisis is there most benefited; and it is benefited mainly because of the invigoration the climate is likely to give to the digestion and nutrition. In those cases of phthisis, however, in which acute inflammatory symptoms are of frequent occurrence, or in which bronchitis exists out of proportion to the local lesion, the dry, bracing quality of the air is too stimulating, and acts as an irritant.

He quotes from Dr. Flagg, of St. Paul, who says that in his experience the patients most likely to be benefited are the purely tuberculous, in whom cuchemia is early developed, and the inflammatory processes are mostly absent; those who have hemoptysis early, and who are described as "relaxed," and wanting in "tone" of mucous surfaces. "Those in advanced stages of phthisis" "often derive positive harm." "The existence of a cavity of any extent, with pus-producing surface, should prevent a patient coming here;" patients in whose lungs there is a large deposit of tubercle rapidly undergoing softening, should not be sent." Those who are subject to inter-current attacks of bronchitis, or who suffer from a sense of "constriction," to use a patient's term, during cold, dry winds, are not very suitable cases.—*Boston Medical and Surgical Journal.*

